# Supplementary material for: Significance of descriptive symptoms and signs and clinical parameters as predictors of neuropathic cancer pain
Source: PLoS One. 2021 Aug 17;16(8):e0252781. doi: 10.1371/journal.pone.0252781 (PMC8370612; doi:10.1371/journal.pone.0252781)
Supplement: S1 Table — (DOCX) [file pone.0252781.s001.docx]

**S1 Table.** Comparison of patients receiving/not receiving chemotherapy and age <65 or ≥65 years.

|  | Chemotherapy | Non-chemotherapy | Total | P-value |
| --- | --- | --- | --- | --- |
| Age <65 years | 1,123 (92.6) | 90 (7.4) | 1,213 (60.6) | <0.001 |
| Age ≥65 years | 630 (79.7) | 160 (20.3) | 790 (39.4) |  |

**Funding Statement**

This research was sponsored by Pfizer Pharmaceuticals Korea Ltd. Ha Yeong Gil, the employee of Pfizer Pharmaceuticals Korea Ltd., contributed in the study design, data analysis, decision to publish, or preparation of the manuscript.
